# Correction to: Natural product pectolinarigenin inhibits osteosarcoma growth and metastasis via SHP-1-mediated STAT3 signaling inhibition

**DOI:** 10.1038/s41419-018-0911-4

**Published:** 2018-09-05

**Authors:** Tao Zhang, Suoyuan Li, Jingjie Li, Fei Yin, Yingqi Hua, Zhouying Wang, Binhui Lin, Hongsheng Wang, Dongqing Zou, Zifei Zhou, Jing Xu, Chengqing Yi, Zhengdong Cai

**Affiliations:** 1 Department of Orthopaedics, Shanghai General Hospital, Shanghai Jiao Tong University School of Medicine, Shanghai, China; 2 The Institute of Cell Metabolism and Disease, Shanghai Key Laboratory of Pancreatic Cancer, Shanghai General Hospital, Shanghai Jiao Tong University School of Medicine, Shanghai, China

Correction to: Cell Death and Disease (2016) 7, e2421; 10.1038/cddis.2016.305; published online 13 October 2016

Since publication of this article, the authors have noticed errors in Fig. 1e (the merge image of control group) and Fig. 5e (Pec. 50 mg/kg group). As a result of the misfiling of the data, incorrect images were inadvertently inserted in Figs. 1e and 5e during figure preparation. The correct figures are given below.

**Fig. 1:**
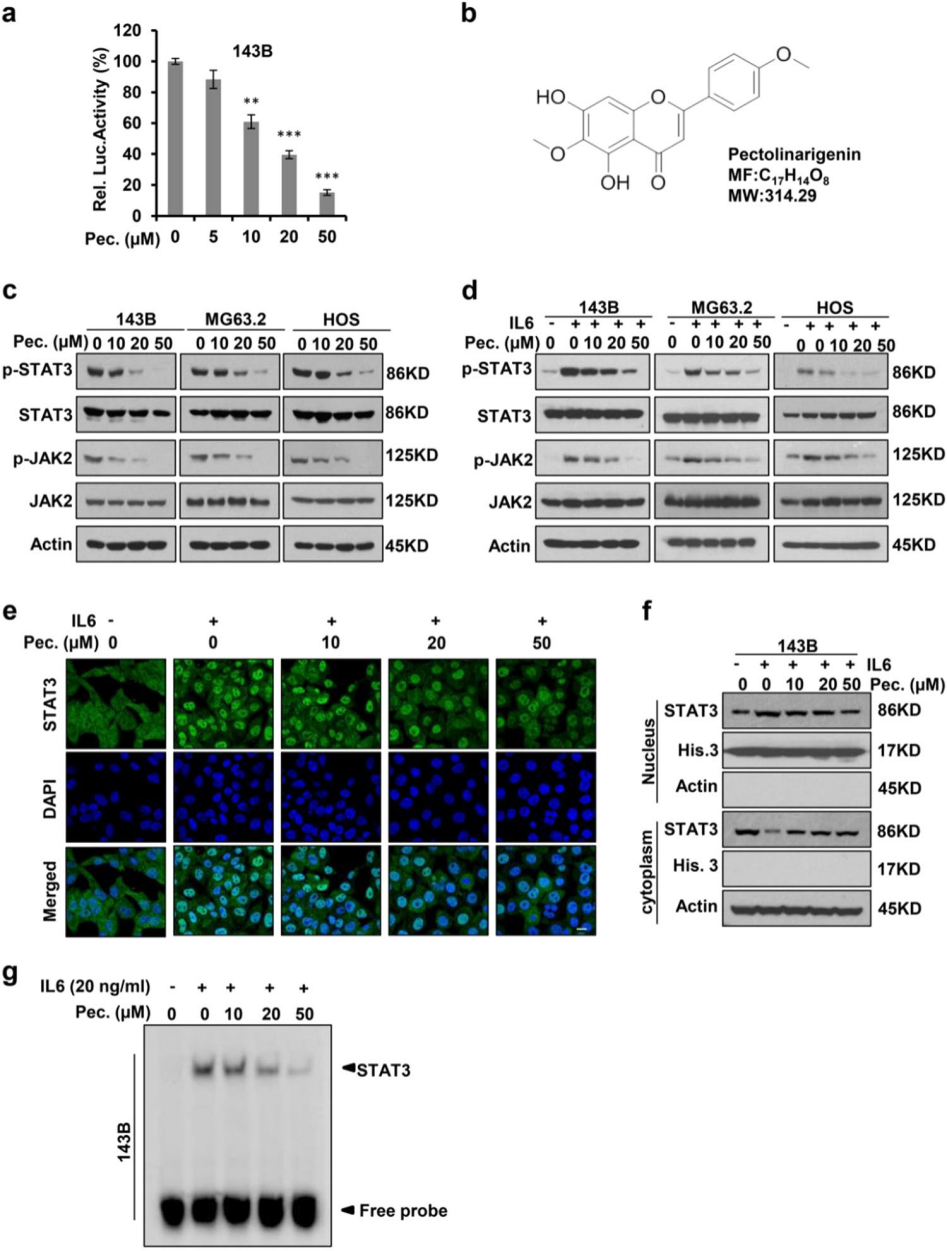
**Pectolinarigenin inhibits STAT3 activity in osteosarcoma. a** 143B cells were transfected with STAT3 Luciferase reporter gene plasmid and treated with different concentrations of pectolinarigenin for 24 h. The results were normalized to the Renilla luciferase activity (**P *<* 0.01; ***P *<* 0.001). **b** Chemical structure of pectolinarigenin. **c** A panel of osteosarcoma cell lines was exposed to the indicated concentrations of pectolinarigenin for 24 h. Cells were then lysed and applied to immunoblotting with the indicated antibodies. Actin was used as an internal control. **d** Osteosarcoma cell lines were pretreated with the indicated concentrations of pectolinarigenin for 24 h and then stimulated with IL6 (20 ng/μL) for 30 min. Whole cell extracts were prepared and subjected to western blot using the indicated antibodies. **e** 143B cells were seeded on gelatin-coated coverslips and pretreated with pectolinarigenin for 24 h followed by stimulating with IL6 (20 ng/μL) for 30 min. The coverslips were examined by a confocal microscopy. Anti-STAT3 antibody (green) was used to locate endogenous STAT3. Cell nuclei were stained with 4′,6-diamidino-2-phenylindole (DAPI). Scale bar, 20 μm. **f** 143B cells were treated with pectolinarigenin for 24 h, and the cytoplasmic and nuclear extractions were subjected to immunoblotting to detect the level of STAT3. **g** 143B cells were pretreated with pectolinarigenin and stimulated with IL6. An EMSA assay was performed to analyze STAT3 DNA-binding activity

**Fig. 5:**
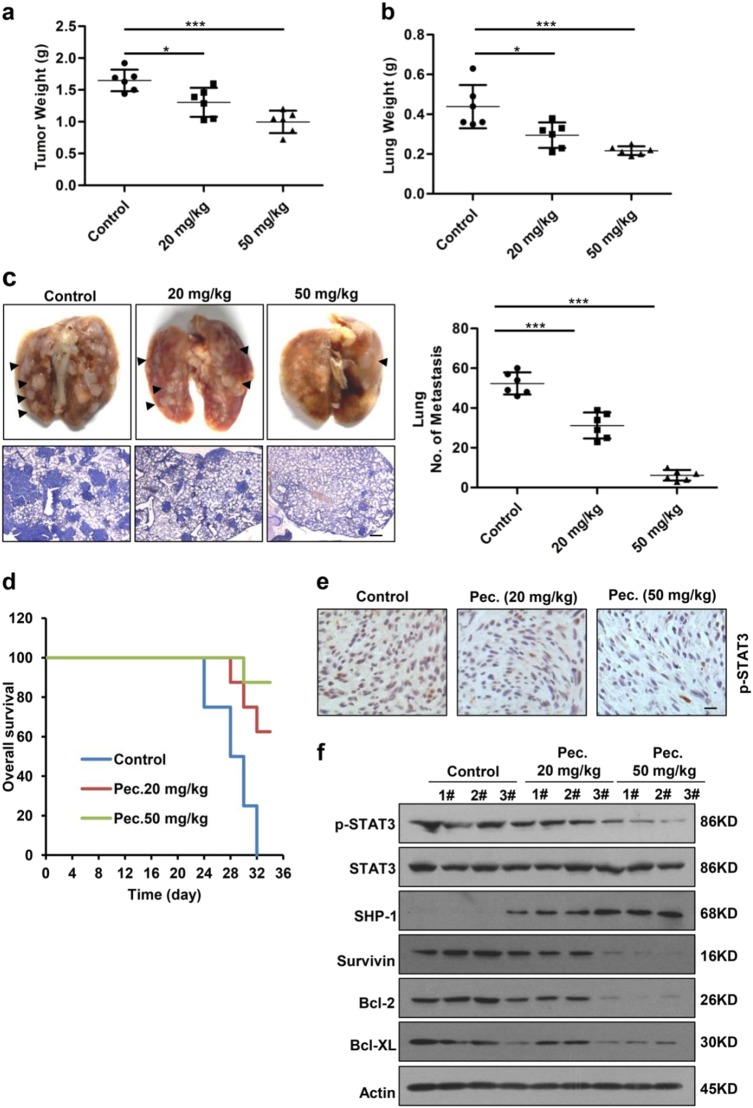
**Pectolinarigenin inhibits tumor growth, metastasis, and prolongs survival in an orthotopic osteosarcoma mouse model. a** 143B cells were injected into the medullary cavity of tibia of the tested mice. 24 days-post pectolinarigenin administration, mice in different groups were sacrificed and the posterior limb with tumors was weighed (*P < 0.05; ***P < 0.001). **b** Lungs in different groups were excised and weighed (*P < 0.05; ***P < 0.001). **c** Lung colonization was visualized by a dissecting microscope and lung metastasis nodules were counted manually (***P < 0.001). Four micrometer sections of lungs were subject to H&E staining (left lower panel). Scale bar, 100 μm. **d** Overall survival rate in the orthotopic osteosarcoma mouse model. **e** Primary tumors were removed, fixed, and paraffin embedded at the end of the experiment. Four micrometer sections were analyzed by IHC using an anti-phospho-STAT3 (Y705) antibody. Scale bar, 100 μm. **f** Primary tumors were lysed and subjected to immunoblotting with indicated antibodies. Actin was used as a loading control

The authors would like to apologize for any inconvenience this may have caused.

